# QuickStats

**Published:** 2014-04-04

**Authors:** FROM THE NATIONAL CENTER FOR HEALTH STATISTICS

**Figure:**
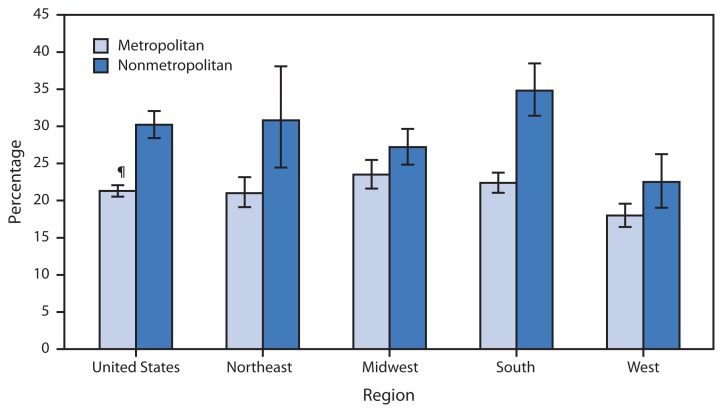
Percentage of Adults Aged ≥65 Years Who Have Lost All Their Natural Teeth,* by Type of Locality^†^ and Region — National Health Interview Survey, United States, 2010–2012^§^ ^*^ Based on response to the question, “Have you lost all of your upper and lower natural (permanent) teeth?” ^†^ The designation of a place of residence as metropolitan or nonmetropolitan is determined by whether the household resides within a metropolitan statistical area, defined as a county or group of contiguous counties that contains at least one urbanized area of ≥50,000 population. Surrounding counties with strong economic ties to the urbanized area are also included. Nonmetropolitan areas do not include a large urbanized area and are generally thought of as more rural. ^§^ Estimates are based on household interviews of a sample of the civilian, noninstitutionalized U.S. population and are derived from the National Health Interview Survey sample adult component. Estimates are age-adjusted using the projected 2000 U.S. population as the standard population and three age groups: 65–74 years, 75–84 years, and ≥85 years. ^¶^ 95% confidence interval.

During 2010–2012, 30% of adults aged ≥65 years living in nonmetropolitan areas had no natural teeth, compared with 21% of those living in metropolitan areas. The percentage of adults aged ≥65 years with no natural teeth was higher in nonmetropolitan areas than in metropolitan areas in all regions of the United States. In both metropolitan and nonmetropolitan areas, the West had the lowest percentage of adults with no natural teeth.

**Sources:** National Health Interview Survey, 2010–2012. Available at http://www.cdc.gov/nchs/nhis.htm.

CDC. Health Data Interactive. Available at http://www.cdc.gov/nchs/hdi.htm.

**Reported by:** Ellen A. Kramarow, PhD, ekramarow@cdc.gov, 301-458-4325.

